# The course of multiple sclerosis rewritten: a Norwegian population-based study on disease demographics and progression

**DOI:** 10.1007/s00415-020-10279-7

**Published:** 2020-10-22

**Authors:** Cecilia Smith Simonsen, Heidi Øyen Flemmen, Line Broch, Cathrine Brunborg, Pål Berg-Hansen, Stine Marit Moen, Elisabeth Gulowsen Celius

**Affiliations:** 1 Department of Neurology, Vestre Viken Hospital Trust, Dronninggata, 3004 Drammen, Norway; 2 Department of Neurology, Hospital Telemark HF, Skien, Oslo, Norway; 3 Department of Neurology, Oslo University Hospital, Oslo, Norway; 4 Institute of Clinical Medicine, University of Oslo, Oslo, Norway; 5 Institute of Health and Society, University of Oslo, Oslo, Norway; 6 Oslo Centre for Biostatistics and Epidemiology, Research Support Services, Oslo University Hospital, Oslo, Norway; 7 MS-Centre Hakadal, Grønvoll, Norway

**Keywords:** Multiple sclerosis, Disease course, Natural history, Epidemiology, Time to EDSS 6

## Abstract

**Objectives:**

Over the past few decades, there has been an improvement in the rate of disability progression in multiple sclerosis (MS) patients, and most studies relate this evolvement to the introduction of disease-modifying therapies. However, several other factors have changed over this period, including access to MRI and newer diagnostic criteria. The aim of this study is to investigate changes in the natural course of MS over time in a near-complete and geographically well-defined population from the south-east of Norway.

**Methods:**

We examined disease progression and demographics over two decades and assessed the effect of disease-modifying therapies using linear mixed-effect models.

**Results:**

In a cohort of 2097 patients, we found a significant improvement in disability as measured by the Expanded Disability Status Scale (EDSS) stratified by age, and the improvement remained significant after adjusting for time on disease-modifying medications, gender and progressive MS at onset. The time from disease onset to EDSS 6 in the total cohort was 29.8 years (95% CI 28.5–31.1) and was significantly longer in patients diagnosed after 2006 compared to patients diagnosed before. There are significant differences between patient demographics, as well as time to EDSS 6, in the near-complete, geographically well-defined population compared to an additional cohort from the capital Oslo and its suburbs.

**Conclusion:**

The natural course of MS is improving, but the improvement seen in disease progression has multifaceted explanations. Our study underlines the importance of completeness of data, relevant timeframes and demographics when comparing different MS populations. Studies on incomplete populations should be interpreted with caution.

**Electronic supplementary material:**

The online version of this article (10.1007/s00415-020-10279-7) contains supplementary material, which is available to authorized users.

## Introduction

In 1989, the median time from onset of multiple sclerosis (MS) to dependence on a walking aid, Expanded Disability Status Scale (EDSS) 6, was 15 years [1]. Several studies have confirmed a delay in the time to reach EDSS 6 over the past two decades [2, 3]. Most studies attribute this delay to the effects of disease-modifying treatments (DMTs) [2, 4, 5].

Much has changed in addition to the introduction of DMTs, including an increase in the global prevalence and incidence of MS [6], revised diagnostic criteria [7] and a possible decrease in the diagnosis of primary progressive MS [8, 9]. Patients more frequently have a less severe disease course and a larger proportion of patients are female [10]. The composition of previous natural history cohorts may, therefore, be of limited relevance as a reference for today’s patients. In addition, recent studies are usually based on large, multicenter databases such as MSBase [11] and national MS registries [12], which rarely represent a complete population [13]. The translatability of these studies to the general MS population is consequently under scrutiny [14]. The aim of this study is to investigate changes in the natural course of MS over time in a complete and geographically well-defined population.

## Materials and methods

### Population

**Fig. 1:**
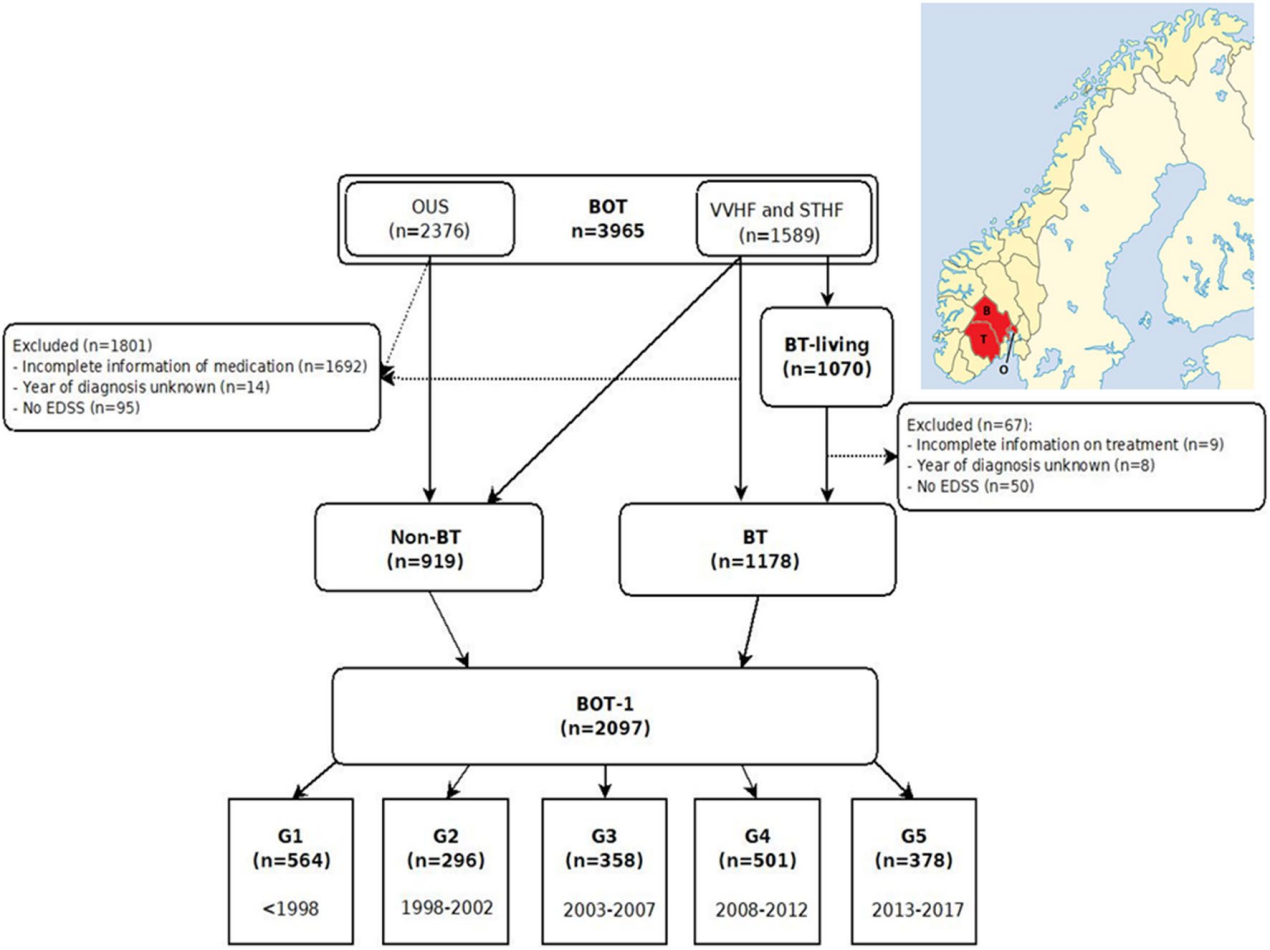
Flow diagram of population with a map of Norway denoting the counties of Buskerud (B), Telemark (T) and Oslo (O) in red (courtesy of Kartverket)

This is a registry-based study. The Buskerud-Oslo-Telemark (BOT) registry contains information on patients diagnosed with MS between 1919 and 2017 in the two regional hospital trusts of Vestre Viken Health Trust (VVHF), with patients mostly from the county of Buskerud, and Telemark Hospital Trust (STHF), as well as in Oslo University Hospital (OUS). These hospitals serve a population of 1.17 million people (490 000, 170 000 and 510 000 respectively) in south-eastern Norway (Fig. 1). The neurological departments at VVHF and STHF serve as the main MS clinics for their populations, and the complete populations from these counties are included. The Oslo population is an incomplete, but a historically unique university MS registry in Norway. Some patients were transferred to another hospital in 2010 due to regional reorganization, and some were lost to follow-up.

All Norwegian citizens have a national identity number that allows for the unique identification of patients. During the study period, every MS patient in Norway and their neurologists had access to all DMTs available since market access in Europe. Almost all patients with MS are followed by neurologists at public hospitals, and there are few neurologists in private practice.

## Methods

We did a search for the ICD-10 diagnosis G35 (MS) in the three hospitals’ electronic medical system in January 2018. All patients were diagnosed according to the prevailing diagnostic criteria at the time [7]. Three experienced MS specialized neurologists examined all medical journals and retrospectively documented information on the disease. They followed predetermined criteria and discussed challenging cases in the group to limit sampling bias.

Disease onset was defined as the time of the first symptom suggestive of MS, type of main symptom based on Kurtzke’s functional systems [15] and whether one or more than one system was affected. We documented time of diagnosis, number of relapses before diagnosis, phenotype, oligoclonal bands and EDSS. Year of death was registered where appropriate.

We documented information on DMTs, including time of start and discontinuation. Only treatments used for at least three months were included. We considered natalizumab, fingolimod, alemtuzumab, mitoxantrone, rituximab and autologous hematopoietic stem cell therapy as highly efficacious DMTs. All induction therapies (mitoxantrone, alemtuzumab, autologous hematopoietic stem cell transplantation) were considered effective from the first dose until the end of observation.

The patients were divided into five subgroups: G1 (diagnosed ≤ 1997), G2 (1998–2002), G3 (2003–2007), G4 (2008–2012) and G5 (2013–2017). G2 is the first group with complete case attainment and the group diagnosed prior to introduction of the first more efficacious DMT, natalizumab, in 2006 [16]. G2 was, thus, used as a comparator for the first group diagnosed after the introduction of more efficacious DMTs and with sufficient time for follow-up, G4. We also compared the populations diagnosed before and after 2006.

### The different cohorts

From the BOT database, we have constructed two main cohorts: (1) “BT-living” is the geographically complete and living population from Buskerud and Telemark, which was used for calculating incidence and prevalence. (2) “BOT-1” contains all living and dead patients from BOT with complete information on diagnosis, treatment and at least one EDSS, regardless of residence. BOT-1 was further divided into five subgroups based on year of diagnosis and was used for the calculations of disease progression (Fig. 1). We have also divided the BOT-1 population into “BT” and “Non-BT”, with the latter only encompassing patients residing outside the geographically well-defined area of Buskerud and Telemark on 1st January 2018 or at time of death. Non-BT mostly includes patients from the Oslo-region.

### Time to EDSS

We considered all recorded EDSS scores, except those assessed within three months after a relapse. If there was no EDSS, but sufficient information available, an EDSS score was constructed. All three researchers obtained Neurostatus certification before data collection [17].

To calculate time to EDSS 6, we included all patients who had reached EDSS 6 and where we knew the year of EDSS 6. We also included those with an updated EDSS in the past 2 years (*n* = 1702) and patients with a known EDSS below four in the last 6 years (*n* = 180). All remaining patients were excluded either due to lack of information on year of onset, uncertainty whether they have reached EDSS 6 or unknown year of reaching EDSS 6. This approach excludes patients with an EDSS 4–5.5 at last clinic visit 3–6 years ago (*n* = 9), who have a high probability of progressing to EDSS 6 in the study period.

### Statistics

All data were collected in EpiData and transferred to IBM SPSS Statistics 25.0 (IBM Corp., Armonk, NY, USA) and Stata 15 (Stata Corp. LLC,College Station, TX, USA). The crude prevalence was estimated based on prevalent MS cases in BT-living on January, 1st 2018. The denominator was the total population of Buskerud and Telemark (*n* = 455 160; Statistics Norway, www.ssb.no/tabell/07459). Annual incidence rates were calculated based on new MS cases between 1998 and 2017, divided by the estimated population at risk. The results are presented as incidence rates (new cases per 100 000 person-years) with 95% confidence intervals (CI). The Wilson 95% CI was calculated for both prevalence and incidence using www.openepi.com. We used the 2013 European standard population for adjustment of incidence and prevalence. Patient years is defined as total number of years from diagnosis to 1st January 2018, death or lost to follow-up.

Differences in continuous variables between two groups were assessed by independent sample *t* test, i.e., BT vs Non-BT and before vs. after 2006. The Chi square test for contingency tables was used to detect associations between categorical variables. One-way ANOVA was used to compare means across the five subgroups. Linear mixed-effects models were used to investigate the EDSS progression between the five groups over the follow-up period, and to account for repeated measures. Age at EDSS was used as the time variable, and all the analyses were stratified on cohort (BOT-1, BT, and Non-BT). In the models, time, time by group interaction and covariates were treated as fixed effects. All models included a random intercept, and an unstructured covariance matrix. The Kaplan–Meier method was used to estimate time to EDSS 6, and Log-Rank test was used to compare groups. Follow-up time was calculated as person-years from time of onset until the date of EDSS 6, date of emigration, death or to January 1st, 2018, whichever occurred first. All *p* values were two-sided and a 5% significance level was used.

### Ethics and data availability

The Regional Ethics Committee in Norway approved this study (REK 2015/670). Specific requests regarding data sharing should be directed to the corresponding author.

## Results

### Prevalence and incidence

In the prevalent MS population of BT-living, 1070 patients were alive and residing in these two counties on date of prevalence, which corresponds to a crude prevalence of 235.1/100 000 (95% CI 221.3–249.7). The prevalence adjusted to the European standard population was 237.4/100 000 (95% CI 223.3–251.6). The gender ratio was 2.1:1 with a female predominance, which did not change significantly over the two decades.

1032 patients were diagnosed between 1998 and 2017. This gives a mean yearly incidence of 12.3/100 000 (95% CI 11.5–13.0). The incidence adjusted to the European Standard population was 12.2/100 000 (95% CI 11.5–13.0). The incidence increased from 10.7/100 000 (95% CI 9.8–11.6) in the period 1998–2009 to 14.4/100 000 (95% CI 13.1–15.6) in the period 2010–2017.

### The population

Of a total of 3965 patients in BOT, comprehensive information on 2097 patients in the BOT-1 cohort was available, which is the equivalence of 27 916 patient years (Fig. 1). G1 lost 30.1% of its population to follow-up, either due to death or relocation. G2 lost 19.9%, G3 lost 12.6%, G4 lost 5.4% and G5 lost 1.3%. We included 93.7% (*n* = 1003) of the complete BT-living population from Buskerud and Telemark. Demographic information on the subgroups is shown in Table 1.

**Table 1:** Patient demographics collectively and in each diagnostic subgroup

	All	G1	G2	G3	G4	G5	*p* value	Effect size (strength of difference between groups)						
		< 1998	1998–2002	2003–2007	2008–2012	2013–2017								
	*n* = 2097	*n* = 564	*n* = 296	*n* = 358	*n* = 501	*n* = 378		G2 vs. G1	G2 vs. G3	G2 vs. G4		G2 vs. G5		Effect size
Sex ratio (% women), *n* = 2097	2.2:1 (68.6)	2.2:1 (69.0)	2.1:1 (67.2)	2.1:1 (67.3)	2.4:1 (70.3)	2.1:1 (68.0)	0.87							
Mean age at onset, years (SD), *n* = 2033	34.6 (10.7)	31.3 (9.2)	34.8 (10.2)	35.5 (10.9)	36.1 (11.3)	36.1 (11.4)	< 0.001	< 0.001	0.9		0.4	0.6		0.03*
Mean age at diagnosis, years (SD) *n* = 2097	39.9 (11.4)	37.1 (10.0)	41.0 (10.2)	41.5 (11.6)	40.7 (12.3)	40.3 (12.3)	< 0.001	< 0.001	0.9		1.0	0.9		0.02*
Mean years onset to diagnosis (SD), *n* = 2033	5.4 (6.8)	5.9 (6.5)	6.3 (7.0)	6.1 (7.3)	4.6 (6.8)	4.2 (6.4)	< 0.001	1.0	1.0		0.006	0.001		0.02*
Progressive MS at onset %, *n* = 1969	11.3	13.7	11.8	12.0	11.2	7.3	0.06							
≥ 2 relapses before diagnosis %, *n* = 1716	69.7	77.2	78.7	71.3	65.7	60.4	< 0.001							0.15^†^
Onset symptoms % *n* = 2001														
Visual	18.8	23.0	16.9	20.8	14.5	18.2	< 0.001							0.15^†^
Motor	20.4	25.1	20.8	20.6	21.1	12.9								
Sensory	34.5	30.9	38.0	33.2	33.0	39.7								
≥ 2 symptoms at onset %, *n* = 1645	32.3	38.7	33.2	29.6	33.5	26.3	0.01							0.09
Mean EDSS at diagnosis (SD), *n* = 1665	2.6 (1.3)	2.9 (1.4)	2.8 (1.4)	2.6 (1.4)	2.5 (1.3)	2.6 (1.3)	< 0.001	1.0	0.2		0.008		< 0.001	0.02*
OCB positive %, *n* = 1935	88.0	83.3	83.7	92.4	93.3	95.4	< 0.001							0.17^†^
Patients never treated %, *n* = 2097	55.9	72.0	48.3	43.3	30.5	17.7	< 0.001							0.39
Deceased patients %, *n* = 2097	189 (9.0)	132 (23.4)	31 (10.5)	17 (4.7)	8 (1.6)	1 (0.3)	< 0.001							0.32^†^

*P* value: Chi square for dichotomous, between groups one-way ANOVA for continuous variables

*Cohen’s classification: 0.01 = small effect, 0.06 = medium effect and 0.14 = large effect

^†^Cramer’s V: 0.1 = small effect, 0.2 = medium effect and 0.3 = large effect

**Fig. 2:**
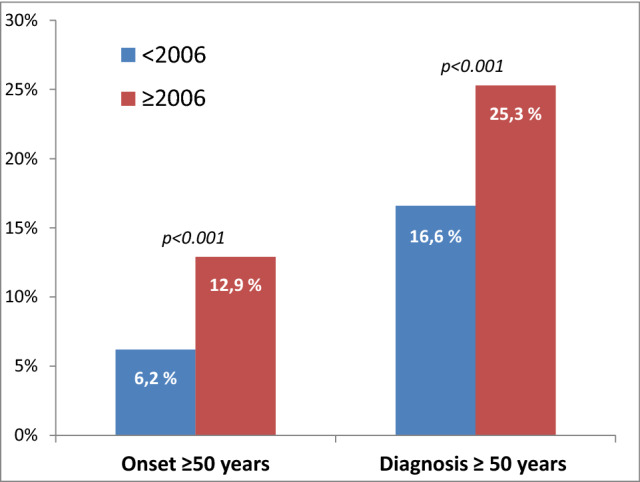
The proportion of older patients (≥ 50 years) at disease onset (*p* < 0.001) and at diagnosis (*p* < 0.001) before and after 2006

We found a significant difference between the proportion of older patients (≥ 50 years) at disease onset (*p* < 0.001) and at diagnosis (*p* < 0.001) before and after 2006 (Fig. 2). Patients who were ≥ 50 years and diagnosed after 2006 had a disease onset 3.0 years later (*p* < 0.001), were diagnosed 1.7 years later (*p* = 0.001) and had a 0.3 point lower EDSS score (*p* = 0.05) compared to those diagnosed before 2006.

### The disease course

**Fig. 3:**
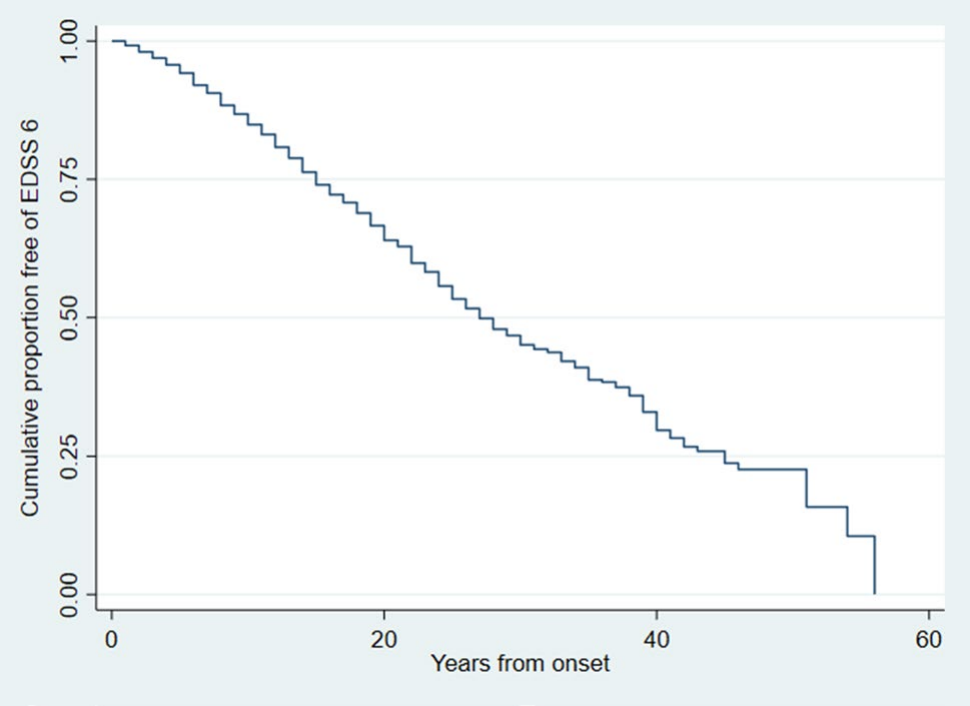
Kaplan–Meier estimate of time from onset to EDSS 6

The mean time from onset to EDSS 6 was 29.8 (95% CI 28.5–31.1) years (Fig. 3). A total of 692 patients (33.0%) had reached EDSS 6, and 1319 (62.9%) had not reached EDSS 6. However, 86 patients who had reached EDSS 6, and 40 patients who had not reached EDSS 6, were excluded due to lack of information on year of onset or year of EDSS 6. Table 2 shows time to EDSS 6 in the different subgroups.

**Table 2:** Means for time to EDSS 6 in five subgroups, patients diagnosed before or after 2006 and BT vs. Non-BT patients

	Years to EDSS 6	95% CI	Reached EDSS 6 %	*p* value
BOT-1	29.8	28.4–31.1	34.5	
BT-living	30.4	28.5–32.4	31.2	
G1 (diagnosed ≤ 1997)	26.7	25.0–28.5	70.5	< 0.001
G2 (1998–2002)	25.9	23.5–28.3	48.5	
G3 (2003–2007)	31.5	28.2–34.8	31.3	
G4 (2008–2012)	36.0	31.6–40.4	12.7	
G5 (2013–2017)	42.8	36.6–49.1	3.7	
Patients diagnosed < 2006	27.6	26.2–29.0	57.8	< 0.001
Patients diagnosed ≥ 2006	35.3	32.0–38.6	12.3	
BT patients	27.3	25.8–28.9	40.4	< 0.001
Non-BT patients	33.8	31.6–35.9	26.7	
Relapsing at onset	32.2	30.7–33.7	27.1	< 0.001
Progressive at onset	13.9	12.1–15.7	70.3	
Progressiv at onset				
G1 (diagnosed ≤ 1997)	12.0	9.6–14.4	96.8	0.04
G2 (1998–2002)	10.8	8.5–13.1	87.9	
G3 (2003–2007)	11.1	8.6–13.6	74.3	
G4 (2008–2012)	19.5	14.0–25.0	46.3	
G5 (2013–2017)	17.2	11.0–17.0	29.6	

*BOT-1* database of all 2097 patients from Buskerud, Oslo and Telemark included in study, BT-living: geographically complete and living population from Buskerud and Telemark, *BT* Buskerud and Telemark, *Non-BT* everyone in BOT-1 who does not reside in Buskerud or Telemark, *CI* confidence interval

**Fig. 4:**
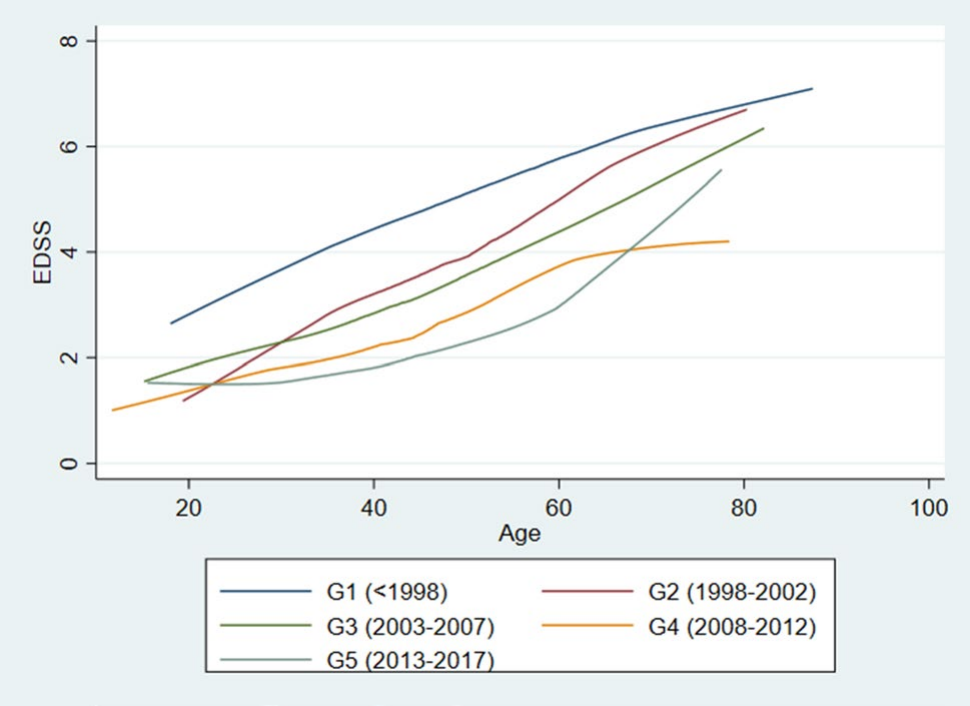
EDSS progression in five diagnostic groups, adjusted for months on all treatment, gender, progressive MS at onset and age at onset

The results from the linear mixed-effects models investigating the EDSS progression over the entire follow-up period are presented in Table 3. Using patients diagnosed between 1998 and 2002 (G2) as a reference, there is a significant delay in EDSS progression by age compared to patients diagnosed between 2008 and 2012 (G4) within all three cohorts (BOT-1: *p* < 0.001, BT: *p* < 0.001, non-BT: *p* = 0.002). After adjusting for months treated with any DMT, age at onset, gender and progressive MS at onset, this improvement remained highly significant (Fig. 4, Table 3). Adjusting for time on highly active DMT did not alter the outcome.

**Table 3:** Results from linear mixed-effects regression analyses (unadjusted and adjusted) of EDSS progression over time

Group	Year of diagnosis	Unadjusted			Adjusted		
		BOT-1			BOT-1		
		(*n* = 13 075 EDSS)			(*n* = 12 713 EDSS)		
		Coef	95% CI	*p* value	Coef	95% CI	*p* value
Age at EDSS		0.144	0.130–0.159	< 0.001	0.148	0.134–0.163	< 0.001
G2	1998–2002	Reference					
Diagnostic group							
G1	< 1998	1.863	1.028–2.552	< 0.001	1.739	0.912–2.567	< 0.001
G3	2003–2007	0.626	− 0.300–1.552	0.19	0.580	− 0.327–1.487	0.21
G4	2008–2012	2.516	1.607–3.424	< 0.001	2.311	1.415–3.207	< 0.001
G5	2013–2017	3.464	2.371–4.558	< 0.001	2.462	1.372–3.552	< 0.001
Diagnostic group by age of EDSS (interaction term)							
G1	< 1998	− 0.02	− 0.036 to − 0.0003	0.05	− 0.021	− 0.040– − 0.003	0.02
G3	2003–2007	− 0.020	− 0.036–0.0004	0.07	− 0.017	− 0.038–0.003	0.09
G4	2008–2012	− 0.072	− 0.092 to − 0.052	< 0.001	− 0.065	− 0.085– − 0.045	< 0.001
G5	2013–2017	− 0.099	− 0.124 to − 0.074	< 0.001	− 0.070	− 0.096– − 0.045	0.001
Intercept		− 3.503	− 4.181 to − 2825	< 0.001	− 1.505	− 2.309–0.701	< 0.001

Adjusted for months spent on all disease-modifying treatment, progressive MS at onset, age at onset and gender. BOT-1: database of all 2097 patients from Buskerud, Oslo and Telemark included in study

*Coef* regression coefficient, *EDSS* expanded disability status scale

We divided the population into “progressive at onset” and “relapsing at onset” and looked at the disease progression in the two separate groups. In our progressive patients, we did not find significant differences between the reference group (G2, diagnosed 1998–2002) and patients diagnosed after 2002, neither before nor after adjustment (treatment, age at onset and gender). Our findings in the relapsing group emulated the population as a whole, both before and after adjustment (supplementary tab1 and supplementary fig 1).

### BT vs. Non-BT

Non-BT patients (*n* = 919) were younger than the geographically defined, near-complete BT population (*n* = 1178) at prevalence date. See Table 4 for demographic differences. The main findings of differences in EDSS progression in the five subgroups remained the same, both before and after adjustment, in both cohorts (supplementary Table 2). There was, however, a significant difference in time from onset to EDSS 6 (*p* = 0.001) (Table 2).

**Table 4:** Comparison of patients in BT and Non-BT patients collectively, as well as before and after 2006

	All patients		*p* value	< 2006		*p* value	2006–2017		*p* value
	BT	Non-BT		BT	Non-BT		BT	Non-BT	
	*n* = 1178	*n* = 1178		*n* = 612	*n* = 440		*n* = 566	*n* = 479	
Sex ratio (% women), *n* = 2097	2.0 (66.2)	2.5 (71.6)	0.01	1.9:1 (65.4)	2:5 (71.8)	0.03	2.0:1 (67.1)	2.5:1 (71.4)	0.14
Age at onset, years (SD), *n* = 2033	35.6 (11.1)	33.2 (10.1)	< 0.001	34.1 (10.3)	31.7 (9.5)	< 0.001	37.2 (11.7)	34.6 (10.5)	< 0.001
Age at diagnosis, years (SD), *n* = 2097	41.3 (11.6)	38.0 (10.9)	< 0.001	40.4 (10.6)	37.1 (10.0)	< 0.001	42.2 (12.5)	38.8 (11.7)	< 0.001
Onset to diagnosis, years (SD), *n* = 2033	5.8 (7.2)	4.8 (6.2)	< 0.001	6.6 (7.2)	5.4 (6.2)	0.005	5.0 (7.2)	4.2 (6.2)	0.07
Progressive MS at onset %, *n* = 1969	12.1	10.3	0.20	13.0	12.5	0.82	11.1	8.4	0.15
≥ 2 relapses before diagnosis %, *n* = 1716	72.7	65.5	0.001	77.9	73.5	0.17	67.5	61.1	0.04
Onset symptoms %, *n* = 2001									
Visual	18.4	19.6	0.15	22.0	19.4	0.08	14.4	19.6	0.11
Motor	22.3	18.2		24.9	20.8		19.5	16.0	
Sensory	34.2	35.0		32.4	33.5		35.9	36.2	
Multiple (≥ 2) symptoms at onset %, *n* = 1645	37.7	25.6	< 0.001	40.7	26.2	< 0.001	33.9	25.1	0.004
Median EDSS at diagnosis (mean), *n* = 1665	2.5 (2.8)	2.0 (2.4)	< 0.001	3.0 (3.0)	2.5 (2.5)	< 0.001	2.5 (2.6)	2.0 (2.3)	< 0.001
OCB pos %, *n* = 1935	88.9	91.7	0.05	82.1	88.3	0.02	94.4	94.5	0.92
Patients never treated %, *n* = 2097	46.9	39.5	0.001	63.2	57.5	0.06	30.2	23.6	0.02
Number of deceased patients (%), *n* = 2097	175 (14.9)	14 (1.5)	< 0.001	161 (26.3)	13 (3.0)	< 0.001	14 (2.5)	1 (0.2)	0.002

*p* value: Independent-sample *t* test for categorical, Chi square for dichotomous

*BT* Buskerud and Telemark, *Non-BT* everyone in BOT-1 who does not reside in Buskerud or Telemark, *SD* standard deviation, *EDSS* expanded disability status score, *OCB pos* oligoclonal bands positive

## Discussion

There is significant delay in the EDSS progression stratified by age between patients diagnosed in 1998–2002 and patients diagnosed in 2008–2012. The difference remained significant after adjusting for the number of months on treatment with DMTs. The delay in disease progression found in this near-complete, geographically well-defined MS population has several explanations, which is supported by our findings that the population demographics have also changed significantly over the past two decades.

Several studies have reported that the natural history of multiple sclerosis is changing, many of which conclude that all or most of this improvement can be attributed to the introduction of DMTs [4, 18–21]. However, the slowing in disease progression seems to have started even before the introduction of DMTs [22].

Meanwhile, prevalence and incidence of MS has increased over the past decades [6]. The prevalence in our population in 2018 was 231.8/100 000, which is an increase from 213.8/100 000 (only Buskerud) in just 4 years [23]. We also observed an increase in the incidence.

There are many reasons for the apparently milder course of disease progression [24]. While this study only spans two decades, our findings demonstrate the rapid change in MS demography over time. We are now diagnosing patients earlier in the disease and our data show that more patients with MS have a less severe disease course. Our findings are significant, and in line with other studies [24–26]. The MS population is changing, and one of the most obvious reasons is the honing of the diagnostic criteria from Schumacher via Poser to McDonald [7, 27–31]. Between 1965 and 2001, the diagnosis of relapsing MS was based on at least two relapses from two or more parts of the CNS. It is now possible to diagnose MS based on one relapse and a single MRI examination. The change in population characteristics due to change in diagnostic criteria is known as the Will Rogers phenomenon, and can lead to perceived improvements in prognosis, even though the outcome of individual patients has not changed [32].

In addition, we are more likely to diagnose older patients. According to the Schumacher criteria, a patient could only be diagnosed with MS if they were younger than 50 years of age [27]. In our population, a quarter of the patients diagnosed in or after 2006 were 50 years or older at the time of diagnosis. We see a steady increase in mean age at onset and diagnosis across the five subgroups. Historically, many older patients would be diagnosed with an undefined neurodegenerative disease before the introduction of MRI [33] or remained undiagnosed [34]. Patients with few symptoms would previously have been assigned a non-specific diagnosis [35]. Our data show that fewer patients have motor symptoms at onset after 2006 compared to historical patients, again supporting the notion of a milder disease [36, 37].

As time passes, the general population has more access to neurologists and MRI machines. The ratio of neurologists per capita in Norway has doubled since 1995 from 4.8/100 000 to 9.5/100 000 in 2017 (data from The Norwegian Doctors’ Union). In 1989, the BMJ published an article by McDonald explicitly advising physicians to only recommend investigating patients presenting with symptoms of MS “when it is clear to the patient that there is something that needs explanation” [38]. Today, we urge primary care doctors to “refer to a neurologist urgently” if they suspect MS, to ensure a prompt diagnosis and initiation of disease-modifying treatment [39].

In addition to the changing demographics of the MS population, there is an increasing amount of data from experimental and clinical trials indicating that exercise can modify the projection of MS [40]. And multiple sclerosis is certainly not the only disease showing a decline in late-life disability. There are documented drops in late-life disability from cardiovascular disease, musculoskeletal conditions, infectious diseases and cancers [41, 42] compared to the 1980s and 1990s, the timing of which corresponds to the improvements in surgical techniques and pharmacological treatments, as well as changes in socioeconomic and lifestyle factors [43]. Smoking has been implicated in the progression of multiple sclerosis [44], but after a concerted effort by governments in the developed world to protect the public from the dangers of tobacco through anti-smoking advertisement, taxes and banning smoking in public spaces, we have seen a decline in the number of smokers in the West [45, 46]. According to Statistics Norway, the percentage of daily smokers above 16 years of age has fallen from 33% in 1998 to 12% in 2018. In addition, numerous observational studies have suggested that there is a correlation between the level of serum vitamin D and disease activity [47], and there has been an increasing focus on vitamin D status supplements [48]. Like us, most studies on disease progression of MS do not register or correct for these factors. Thus, the possible influence of these factors on disease progressions remains unknown.

All these factors combined evidently cause a reduction in time from onset to diagnosis and less severe disease. Subsequently, historical cohorts have likely been enriched with severe MS cases. An illustrative example of this is the changing characteristics of the placebo patients in randomized controlled trials [49]. The annualized relapse rate in the first year after treatment for the placebo groups in the AFFIRM trial from 2006 [16] and CLARIFY from 2010 [50] was 0.81 and 0.33 respectively. The first interferon trials reported a 30% reduction in annual relapse rate (ARR) compared to placebo [51], and the newer disease-modifying drugs halved the ARR compared to placebo 10 years later [16, 52]. However, as our findings demonstrate, MS populations 10 years apart are not the same and real-world studies comparing older drugs in historical cohorts with newer drugs should be interpreted with caution [53].

Contrary to the majority of other studies on disease course, we have limited selection bias and know who is missing and why. The prevalent population includes all MS patients in the two counties of Buskerud and Telemark (BT), and is near-complete. There will always be patient leakage for one reason or another, but this is negligible in our study, due to geographical considerations and the setup of the health care system. To our knowledge, this is the most complete MS registry of a geographically well-defined population.

We did not find a similarly significant improvement in EDSS progression stratified by age in the progressive only group, though we did find a significant improvement in time to EDSS 6 for progressive patients in the five subgroups. However, these numbers are small and should be interpreted with caution. And although the changes in EDSS despite DMTs remained, in most part, the same between the complete BT population and the non-BT population, we did find a difference in time to EDSS 6. In addition, there are some significant differences as the non-BT population includes the Oslo region biased towards younger, more educated, wealthier and more treated patients than the rest of our population. This is in itself an important finding and raises concern as to the generalizability of non-population-based studies. The incomplete non-BT was, therefore, interesting for comparison since many heavily cited papers that are applauded for their size either have a population stemming from tertiary, often university-based MS clinics in larger cities [11, 18], or incomplete populations [2, 3].

The retrospective study design entails investigator bias and missing data. This was ameliorated by the fact that only three MS specialists have included data based on a mutually accepted manual. However, even in our near-complete, prevalent BT population, we had to exclude 78 patients due to missing information on date of diagnosis, EDSS or treatment. The non-BT population was an arbitrary collection of patients in the Oslo region, not unlike patients in large registries. In addition, we cannot rule out a small amount of informative censoring in which patients diagnosed before 1998 may be lost to follow-up. These patients are either severely disabled or have a very “benign” disease. We believe this to be an insignificant number considering the nature of our health care system. There is some uncertainty with regards to time to EDSS 6 as we only included 89.9% of the BOT-1 population and 86.6% of the geographically complete prevalent population in our Kaplan Meier analysis. This may overestimate the time to EDSS 6 slightly, though our case ascertainment is still better than most registry-based studies on time to EDSS 6.

## Conclusion

Our retrospective study over two decades of a modern, well-defined and thoroughly clinically characterized Norwegian MS population shows a significant delay in the EDSS progression stratified by age. However, significant improvement in disability is seen in all MS patients diagnosed after 2006 compared to patients diagnosed before, even after correcting for the effect of DMTs. Our findings emphasize the importance of taking the alterations in demographics, timeframe and completeness of population data and overall treatment into account when looking at changes in disease progression over time. Thus, historical cohorts are unsuitable for comparison in MS studies, and caution is required when comparing different MS populations.

## Electronic supplementary material

Below is the link to the electronic supplementary material.

Supplementary file1 (PDF 109 kb)

Supplementary file2 (PDF 219 kb)

## Data Availability

Not applicable.
