# Supplementary material for: The course of multiple sclerosis rewritten: a Norwegian population-based study on disease demographics and progression
Source: J Neurol. 2020 Oct 22;268(4):1330–41. doi: 10.1007/s00415-020-10279-7 (PMC7990804; doi:10.1007/s00415-020-10279-7)
Supplement: Supplementary file 1 — Supplementary file1 (PDF 109 kb) [file 415_2020_10279_MOESM1_ESM.pdf]

Supplementary tables 1 and 2

The Course of Multiple Sclerosis Rewritten: A Norwegian Population Based Study on Disease Demographics and Progression

Cecilia Smith Simonsen[1,3,4], Heidi Øyen Flemmen[2,5], Line Broch[1,3,4], Cathrine Brunborg[6], Pål Berg-Hansen[3], Stine Marit Moen[7], Elisabeth Gulowsen Celius[3,4]

[1]*Department of Neurology, Vestre Viken Hospital Trust, Drammen, Norway*
[2]*Department of Neurology, Hospital Telemark HF, Skien, Norway*
[3]*Department of Neurology, Oslo University Hospital, Norway*
[4]*Institute of Clinical Medicine, University of Oslo, Norway*
[5]*Institute of Health and Society, University of Oslo, Norway*
[6]*Oslo Centre for Biostatistics and Epidemiology, Research Support Services, Oslo University Hospital, Norway*
[7]*MS-Centre Hakadal, Norway*

Corresponding author:

Cecilia Smith Simonsen (MD):

Department of Neurology, Vestre Viken Hospital Trust, Dronninggaten, 3004 Drammen, Norway

Email: cecsim@vestreviken.no

Phone number: +47 98 67 32 56

		Unadjusted						Adjusted					
		BT			Non-BT			BT			Non-BT		
		(n=7991 EDSS)			*(n=5084 EDSS)*			*(n=7782 EDSS)*			*(n=4931 EDSS)*		
**Group**	**Year of diagnosis**	**Coef.**	**95% CI**	***p***	**Coef.**	**95% CI**	***p***	**Coef.**	**95% CI**	***p***	**Coef.**	**95% CI**	***p***
**Age at EDSS**		0.157	0.128 - 0.176	<0.001	0.124	0.101 - 0.146	<0.001	0.161	0.141 - 0.180	<0.001	0.127	0.104 - 0.150	<0.001
**G2**	1998-2002	*Reference*						*Reference*					
**Diagnostic group**													
**G1**	<1998	1.966	0.841 - 3.090	0.001	1.632	0.401 - 2.864	0.009	1.709	0.593 - 2.826	0.003	1.527	0.309 - 2.745	0.01
**G3**	2003-2007	0.821	-0.445 - 2.088	0.20	0.195	-1.145 - 1.536	0.78	0.766	-0.472 - 2.004	0.23	0.161	-1.156 - 1.477	0.81
**G4**	2008-2012	3.305	2.068 - 4.542	<0.001	1.380	0.056 - 2.704	0.04	3.153	1.934 - 4.372	<0.001	1.107	-0.198 - 2.413	0.10
**G5**	2013-2017	3.948	2.414 - 5.481	<0.001	2.707	1.170 - 4.245	0.001	2.628	1.098 - 4.158	0.001	2.029	0.506 - 3.552	0.009
**Diagnostic group by age of EDSS (interaction term)**													
**G1**	<1998	-0.014	-0.038 - 0.010	0.20	-0.022	-0.049 - 0.006	0.12	-0.016	-0.040 - 0.008	0.20	-0.025	-0.052 - 0.003	0.08
**G3**	2003-2007	-0.025	-0.052 - 0.002	0.07	-0.009	-0.400 - 0.021	0.54	-0.023	-0.050 - 0.003	0.09	-0.006	-0.036 - 0.024	0.71
**G4**	2008-2012	-0.089	-0.116 - -0.063	<0.001	-0.048	-0.078 - -0.017	0.002	-0.084	-0.110 - 0.057	<0.001	-0.036	-0.066 - -0.006	0.02
**G5**	2013-2017	-0.110	-0.143 - -0.076	<0.001	0.082	-0.118 - -0.046	<0.001	-0.076	-0.111 - 0.042	<0.001	-0.057	-0.093 - -0.020	0.002
**Intercept**		-4.039	-4.94 - -3.135	<0.001	-2.621	3.635 - 1.608	<0.001	-1.655	-2.739 - -0.570	0.003	-1.095	-2.271 - 0.081	0.07

Supplemetary table 1: *Results from linear mixed-effects regression analyses (unadjusted and adjusted) of EDSS progression over time by diagnostic groups stratified on cohort. Adjusted for months spent on all disease modifying treatment, progressive MS at onset, age at onset and gender. Non-BT: everyone in BOT-1 who does not reside in Buskerud or Telemark. Coef.: regression coefficient, EDSS: expanded disability status scale*

		Unadjusted						Adjusted					
		**Progressive at onset** *(n=1317)*			**Relapsing at onset** *(n=10 949)*			**Progressive at onset** *(n=1256)*			**Relapsing at onset** *(n=10 751)*		
**Group**	**Year of diagnosis**	**Coef.**	**95% CI**	***p***	**Coef.**	**95% CI**	***p***	**Coef.**	**95% CI**	***p***	**Coef.**	**95% CI**	***p***
**Age at EDSS**		0.188	0.145 - 0.231	<0.001	0.137	0.121 - 0.153	<0.001	0.198	0.157 - 0.240	<0.001	0.140	0.123 - 0.156	<0.001
**G2**	1998-2002	*Reference*						*Reference*					
**Diagnostic group**													
**G1**	<1998	3.399	0.274 - 6.524	0.033	1.351	0.447 - 2.256	0.003	3.077	0.103 - 6.051	0.043	1.291	0.395 - 2.186	0.005
**G3**	2003-2007	-2.628	-6.145 - 0.890	0.143	0.887	-0.086 - 1.860	0.074	-2.019	-5.310 - 1.271	0.229	0.737	-0.219 - 1.693	0.131
**G4**	2008-2012	-2.572	-6.145 - 1.002	0.158	3.073	2.121 - 4.025	<0.001	-1.907	-5.319 - 1.506	0.273	2.659	1.719 - 3.599	<0.001
**G5**	2013-2017	1.482	-3.767 - 6.730	0.580	3.730	2.597 - 4.864	<0.001	-2.000	-6.813 - 2.812	0.415	2.657	1.512 - 3.782	<0.001
**Diagnostic group by age of EDSS (interaction term)**													
**G1**	<1998	-0.054	-0.107 - -0.0004	0.048	-0.009	-0.029 - 0.012	0.40	-0.057	-0.109 - -0.005	0.031	-0.010	-0.031 - 0.010	0.325
**G3**	2003-2007	0.024	-0.036 - 0.084	0.437	-0.023	-0.045 - -0.001	0.038	0.022	-0.036 - 0.080	0.456	-0.020	0.042 - 0.002	0.082
**G4**	2008-2012	0.015	-0.046 - 0.077	0.628	-0.084	-0.106 - -0.063	<0.001	0.014	-0.046 - 0.074	0.654	-0.073	-0.095 - 0.051	<0.001
**G5**	2013-2017	-0.070	-0.159 - -0.019	0.124	-0.105	0.131 - -0.078	<0.001	0.008	-0.076 - 0.091	0.855	-0.075	-0.101 - -0.047	<0.001
**Intercept**		-4.679	-7.189 - -2.168	<0.001	-3.278	-3.998 - -2.557	<0.001	0.920	-1.782 - 3.622	0.505	-1.460	-2.311 - -0.608	0.001

Supplememtary table 2*: Results from linear mixed-effects regression analyses (unadjusted and adjusted) of EDSS progression over time by diagnostic groups stratified on cohort in progressive and relapsing patients Adjusted for months spent on all disease modifying treatment, age at onset and gender, Coef.: regression coefficient, EDSS: expanded disability status scale.*
