# Supplementary material for: The course of multiple sclerosis rewritten: a Norwegian population-based study on disease demographics and progression
Source: J Neurol. 2020 Oct 22;268(4):1330–41. doi: 10.1007/s00415-020-10279-7 (PMC7990804; doi:10.1007/s00415-020-10279-7)
Supplement: Supplementary file 2 — Supplementary file2 (PDF 219 kb) [file 415_2020_10279_MOESM2_ESM.pdf]

Supplementary figure 1

The Course of Multiple Sclerosis Rewritten: A Norwegian Population Based Study on Disease Demographics and Progression

Cecilia Smith Simonsen[1,3,4], Heidi Øyen Flemmen[2,5], Line Broch[1,3,4], Cathrine Brunborg[6], Pål Berg-Hansen[3], Stine Marit Moen[7], Elisabeth Gulowsen Celius[3,4]

[1]Department of Neurology, Vestre Viken Hospital Trust, Drammen, Norway
[2]Department of Neurology, Hospital Telemark HF, Skien, Norway
[3]Department of Neurology, Oslo University Hospital, Norway
[4]Institute of Clinical Medicine, University of Oslo, Norway
[5]Institute of Health and Society, University of Oslo, Norway
[6]Oslo Centre for Biostatistics and Epidemiology, Research Support Services, Oslo University Hospital, Norway
[7]MS-Centre Hakadal, Norway

Corresponding author:

Cecilia Smith Simonsen (MD):

Department of Neurology, Vestre Viken Hospital Trust, Dronninggata, 3004 Drammen, Norway

Email: cecsim@vestreviken.no

Phone number: +47 98 67 32 56

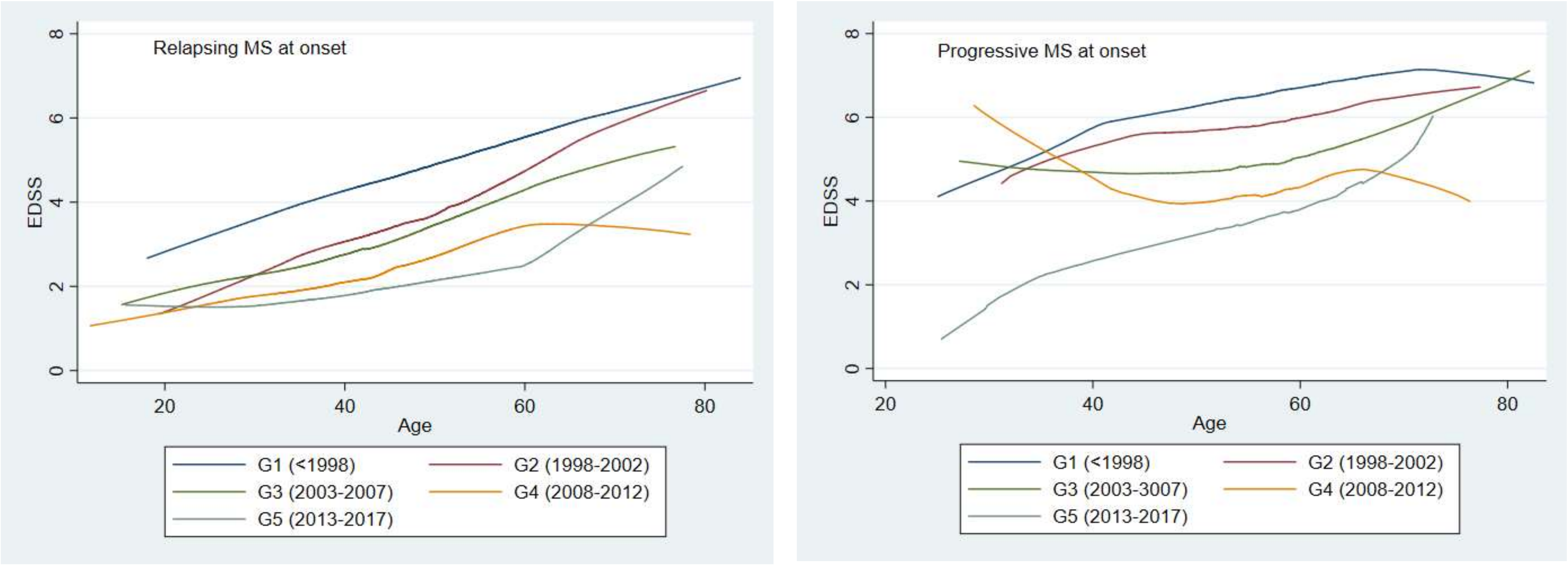

***Supplementary figure**: EDSS progression in five diagnostic groups, adjusted for months on all treatment, gender, progressive MS at onset and age at onset in relapsing MS at onset (left) and progressive MS at onset (right)*
